# Uniqueness of the *Gossypium mustelinum* Genome Revealed by GISH and 45S rDNA FISH

**DOI:** 10.1111/jipb.12084

**Published:** 2013-07-18

**Authors:** Qiong Wu, Fang Liu, Shaohui Li, Guoli Song, Chunying Wang, Xiangdi Zhang, Yuhong Wang, David Stelly, Kunbo Wang

**Affiliations:** 1 Cotton Research Institute, the Chinese Academy of Agricultural Sciences/National Key Laboratory of Cotton BiologyAnyang, 455000, China; 2 Haikou Experimental Station, Chinese Academy of Tropical Agricultural SciencesHaikou, 570102, China; 3 Department of Soil and Crop Sciences, Texas A&M UniversityCollege Station, Texas, 77843-2474, USA

**Keywords:** Chromosomes, cotton, *Gossypium mustelinum*, *in situ* hybridization, nucleolar organizer region, genome

## Abstract

*Gossypium mustelinum* ((AD)_4_) is one of five disomic species in *Gossypium*. Three 45S ribosomal DNA (rDNA) loci were detected in (AD)_4_ with 45S rDNA as probe, and three pairs of brighter signals were detected with genomic DNA (gDNA) of *Gossypium* D genome species as probes. The size and the location of these brighter signals were the same as those detected with 45S rDNA as probe, and were named GISH-NOR. One of them was super-major, which accounted for the fact that about one-half of its chromosome at metaphase was located at chromosome 3, and other two were minor and located at chromosomes 5 and 9, respectively. All GISH-NORs were located in A sub-genome chromosomes, separate from the other four allopolyploid cotton species. GISH-NOR were detected with D genome species as probe, but not A. The greatly abnormal sizes and sites of (AD)_4_ NORs or GISH-NORs indicate a possible mechanism for 45S rDNA diversification following (AD)_4_ speciation. Comparisons of GISH intensities and GISH-NOR production with gDNA probes between A and D genomes show that the better relationship of (AD)_4_ is with A genome. The shortest two chromosomes of A sub-genome of *G. mustelinum* were shorter than the longest chromosome of D sub-genome chromosomes. Therefore, the longest 13 chromosomes of tetraploid cotton being classified as A sub-genome, while the shorter 13 chromosomes being classified as D sub-genome in traditional cytogenetic and karyotype analyses may not be entirely correct.

Wu Q, Liu F, Li S, Song G, Wang C, Zhang X, Wang Y, Stelly D, Wang K (2013) Uniqueness of the *Gossypium mustelinum* genome revealed by GISH and 45S rDNA FISH. *J. Integr. Plant Biol.* 55(7), 654–662.

## Introduction

The *in situ* hybridization (ISH) technique, developed more than 30 years ago (Gall and Pardue 1969; John et al. 1969), has proved to be a powerful technique in cytological biology studies. Jiang and Gill (1994) stressed florescent *in situ* hybridization (FISH) as one of the most important techniques in plant molecular cytogenetic research, because it allows DNA sequences to be mapped directly on chromosomes (Cheng et al. 1998, 2001; Dai et al. 2005; Liu et al. 2006; Jiang and Bikram 2006; Tang et al. 2007). Genomic *in situ* hybridization (GISH) is one of the most effective methods to obtain integrated information of DNA biology on genomic chromosomes, and is therefore powerful in its ability to provide data on genomic evolution and specific relationships (Tan et al. 2006; Liu et al. 2007; Zhou et al. 2008). FISH has been well applied in *Gossypium* (Crane et al. 1993; Hanson et al. 1995, 1995; Cronn et al. 1996; Ji et al. 1997, 1999a, 1999b, 2007; Zhao et al. 1998; Wang et al., 2001b, 2004, 2006, 2007; Liu et al. 2005; Guan et al. 2008; Wu et al. 2008). We recently reported GISH-NOR in AD cultivated cottons (Liu et al. 2005; Song et al. in 2013). Primarily, recent FISH experiments on (AD)_4_ with ribosomal DNA (rDNA) as probes surprised us with abnormal hybridization signals.

Phylogenetic analyses have demonstrated that allopolyploid (2*n* = 4*x* = 52) cottons have radiated into three lineages since their formation, collectively comprising five species: *Gossypium hirsutum* ((AD)_1_), *G. barbadense* ((AD)_2_), *G. tomentosum* ((AD)_3_), *G. mustelinum* ((AD)_4_), and *G. darwinii* ((AD)_5_). The extant A and D genome species are most closely related to their diploid (2*n* = 2*x* = 26) progenitors (Wendel 1989; Wendel and Percy 1990; DeJoode and Wendel 1992; Wendel et al. 1994, 1995; Cronn et al. 1996; Small and Wendel 1999). *G. mustelinum* is basal among the five AD species and the sole descendent of one branch of the earliest divergence within the AD lineages (Small et al. 1998). Internal transcribed spacer (ITS) sequences of (AD)_4_ have concerted to an A genome-like sequence, while the ITS sequences of the remaining AD cottons have concerted to a D genome-like sequence (Wendel et al. 1995). *G. mustelinum* was the most poorly understood among the *Gossypium* AD species, and the majority of effort in studies on AD cottons focused on (AD)_1_ and (AD)_2_ for their economic importance (Wendel et al., 1994). However, the species has great potential for use as a basically non-substitutable material for studies of speciation and evolution of *Gossypium*, particularly disomic cottons, and is a useful genetic resource to construct mapping populations and to improve cotton cultivars (Wendel et al. 1994, 1995; Wendel and Cronn 2003; Westengen et al. 2005). However, to this point, little is understood about the species in terms of molecular cytogenetics, especially for FISH and GISH. Therefore, in this study, we focused on detailed information on (AD)_4_ FISH and GISH related to possible diploid progenitors, as well as on a greater understanding of their AD genome natural status.

## Results

### NORs generated by 45S rDNA

**Figure 1:**
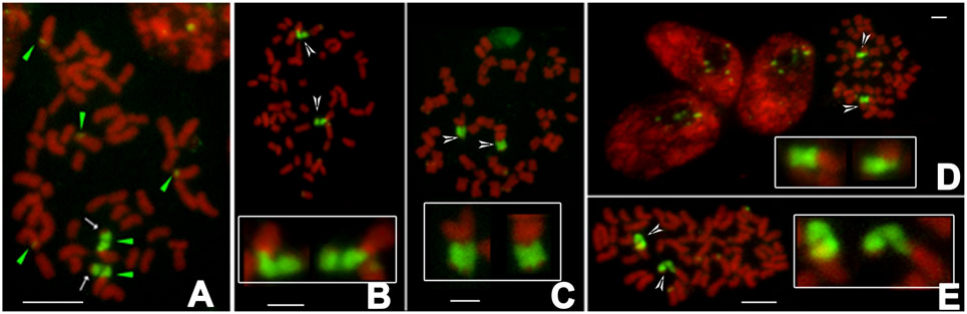
FISH of (AD)_4_ with 45s rDNA (arrows and yellow–green) as probe while counterstained with propidium iodide (PI). (A–E) FISH of mitotic metaphases (AD)_4_ with 45s rDNA as probe, being counterstained with PI. (B–E) Show the super-major 45s rDNA loci with enlarged parts bearing chromosomes. (D) FISH of mitotic interphase (AD)_4_ with 45s rDNA as probe; bright signals were mainly clustering in the nucleoli.

FISH of 45S rDNA revealed a maximum of six major hybridization signals per cell, with the number ranging from two to six (Table2). Signals from FISH of 45S rDNA to mitotic interphase chromatin (Figure 1D) were clustered near or colocalized with the nucleoli, as expected of nucleoalar organizer regions (NORs). The six FISH signals were distributed on six chromosomes that in most cells are collectively identifiable as three distinct pairs of homologs. Features of the chromosomes and signals varied in a correlated manner and reflected the disomic nature of the species.

Of the three pairs of *G. mustelinum* 45S rDNA FISH sites, one was very large, and two were much smaller (Figure 1A–E). Following terminology used by Hanson et al. (1996), the largest site could be classified as “major” or perhaps “super-major,” and the two minor sites as “intermediate,” or perhaps even “minor”. The largest 45S rDNA site accounted for about one-half of the respective chromosomes at metaphase. The super-major NOR exhibited an extremely distinctive distribution of the FISH signal, in that the signal of its middle region was absent or greatly diminished. Flanked by brightly fluorescing segments, this middle region of the NOR was similar in appearance, at least superficially, to a centromere. There was no spread in which a centromere was clearly recognizable at any other location in this chromosome, so it remains quite possible, if not probable, that the super-major NOR flanks the centromere of this *G. mustelinum* chromosome. The two small NORs were located near the ends of the short arms of the respective chromosomes. One of them, however, was clearly closer to its telomere than the other (Figure 1A–E).

### NORs generated by genomic DNA of D genome species

**Figure 2:**
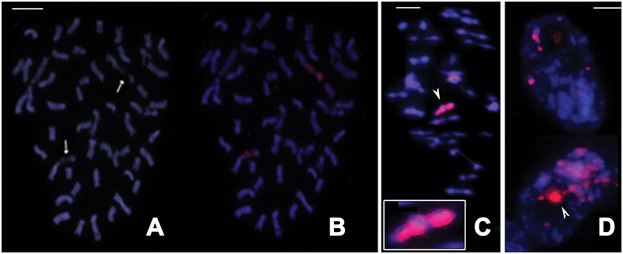
GISH of (AD)_4_, mitotic chromosomes with diploid D genome species gDNA as probe while counterstained with DAPI. (A) Chromosomes of (AD)_4_ counterstained with DAPI; white arrows show two satellites. (B) GISH of (AD)_4_ with D_3-d_ gDNA as probe and salmon sperm DNA as block. (C) GISH of (AD)_4_ meiotic metaphase I with D5 gDNA as probe and A_1-a_ gDNA as block. Partially enlarged detail of C shows special morphology of the super-major GISH-NOR bearing a bivalent chromosome. (D) GISH of (AD)_4_ mitotic interphase with D5 gDNA as probe while salmon sperm DNA as block. GISH-NOR signals were mainly clustered in nucleoli.

Early on we reported the GISH-NOR in *Gossypium*, in the case of which NORs could also be generated by genomic DNA (gDNA) from species such as *G. raimondii* (D_5_) and *G. thurberi* (D_1_) as probes to hybridize to mitotic chromosomes of some allotetraploid or diploid species, such as *G. hirsutum* ((AD)_1_), *G. herbaceum* var. *africanum* (A_1-a_) (Liu et al. 2005), and *G. barbadense* ((AD)_2_) (Song et al. in 2013). Because of the super-major NOR in (AD)_4_, it would be worthwhile to test whether or not GISH-NOR in (AD)_4_ is the same as those in other allotetraploids. Figure 2A–D clearly shows that the GISH-NORs were also generated by gDNA probes from *Gossypium* D genome species, including (at the least) D_5_, D_1_, and *G. davidsonii* (D_3-d_) examined in this study. Like in (AD)_1_ and (AD)_2_, the GISH-NOR numbers and sites in (AD)_4_ were identical to or similar as those generated by 45S rDNA. Also very interestingly, there was one pair of very big GISH-NORs in (AD)_4_, taking the same number and locus as the super-major NOR, which was proved through both mitotic (Figure 2A, C) and meiotic spreads (Figure 2B). Furthermore, the hybridization signal of the super-major GISH-NOR from both mitotic and meiotic metaphases even exhibited great similarity in its morphology.

When comparing the diamidino-2-phenylindole (DAPI) images (Figure 2A) with hybridization images (Figure 2B) with D_3-d_ gDNA as probe, the super-major NOR was natural for the species *G. mustelinum* because two respective images show the same NOR constructions in the two single chromosomes (arrows in Figure 2A and hybridization signals in Figure 2B). The one super-major NOR or super-major GISH-NOR in the species was located in the short arms of a pair of homologous chromosomes. Because it was distributed in such a large area in the chromosome, its locus or region flanked both the centromere and the sub-terminal, which was largely different from the two cultivated disomic cottons (AD)_1_ and (AD)_2_, in which NORs or GISH-NORs were all terminal types (Liu et al. 2005; Song et al. in 2013).

### Comparison of gDNA GISHs between probes by A and D genome species

**Figure 3:**
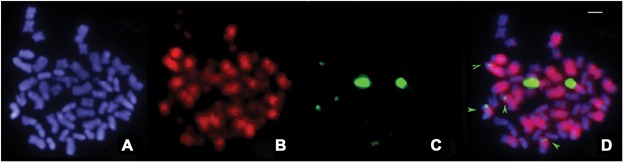
Dual-GISH of (AD)_4_ mitotic metaphase chromosomes with A2 gDNA and 45s rDNA as probes and salmon sperm DNA as blocks. (A) Chromosomes were counterstained with DAPI. (B) GISH signals with A2 gDNA as probe (orange–red). (C) FISH signals with 45s rDNA as probe (yellow–green). (D) A composite picture of Figure 3A–C, arrows show minor signals of 45s rDNA.

**Figure 4:**
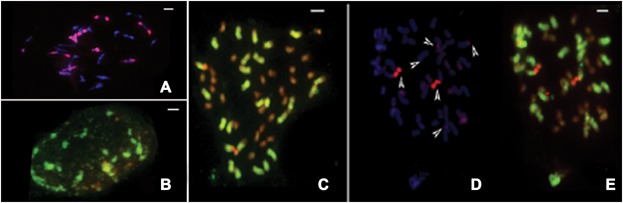
GISH of (AD)_4_ diploid A genome species gDNA as probe and dual-GISH of (AD)_4_ diploid A and D genome species gDNA as probes. (A) GISH of meiotic metaphase I (AD)_4_ with A_1-a_ gDNA as probe and D_3-d_ gDNA as block, showing signals of 13 II of (AD)_4_ (orange–red) and 13 IID of (AD)_4_ counterstained with DAPI. (B) GISH image of mitotic interphase (AD)_4_ with A2 gDNA as probe (yellow–green) and salmon sperm DNA as block. (C) GISH of (AD)_4_ mitotic metaphase chromosomes with A2 gDNA as probe (yellow–green) and salmon sperm DNA as block. Chromosomes were counterstained with PI (orange–red). (D) GISH of (AD)_4_ with D_3-d_ gDNAs as probe (orange–red). Arrows show GISH-NOR and signals in A sub-genome chromosomes. (E) GISH of same (AD)_4_ chromosomes in D with A2 gDNAs as probe (yellow–green) and counterstained with PI, but no yellow–green dominant GISH-NORs were detected.

Figure 3 illustrates dual-FISH of (AD)_4_ mitotic chromosomes with gDNA of *G. arboreum* (A_2_) and 45S rDNA as probes. 45S rDNA (Figure 3C, D) clearly showed two NORs and one super-major NOR, while FISH with A_2_ genome as probe (Figure 3B, D) did not show any NOR or GISH-NOR hybridization signals. This case was also clearly illustrated by the genome FISH images of A_1_ (*G. herbaceum*) and its wild species (A_1-a_) in both meiotic metaphase I (Figure 4A) and mitotic interphase spreads (Figure 4B), which did not show any dotted or very bright hybridization signals in Figure 3D or Figure 2C. Therefore, also for (AD)_4_, GISH-NORs were specific to D genome species, which was highly in line with the two cultivated disomic cottons.

One point that should be emphasized is that the hybridization signals of (AD)_4_ with gDNAs from A genome species (A_1_ and A_2_; Figure 3; Figure 4A–C, E) as probes were much better in intensity coverage and dominance than those from D genome species (at least for D_5_, D_1_, D_3-d_ and D_6_; Figures 2, 4D), which was highly similar to the case for (AD)_1_ and (AD)_2_. This also indicates that A genome is more closely related to allotetraploid cottons than D genome. Or stated in terms of species evolution or allopolyploid speciation, A genome contributed more than D genome.

Sub-genome locations of the NORs or GISH-NORs in (AD)_4_ were a focal point in this study. We noted that all hybridization signals in (AD)_4_ mitotic chromosomes, generated by either gDNA (from both A genome and D genome) or 45S rDNA (Figures 2B, 3E, 4C–E), were located in A sub-genome chromosomes, which was largely different from (AD)_1_ and (AD)_2_. In late two disomic cottons, one NOR or GISH-NOR was located in A sub-genome, and the other two in D sub-genome, which indicates that NORs distribute mainly to D sub-genome. Our recent research (data unpublished) demonstrated that NORs or GISH-NORs in two other wild disomic cottons, *G. tomentosum* ((AD)_3_) and *G. darwinii* ((AD)_5_), were also distributed like (AD)_1_ and (AD)_2_. The sole A sub-genomic distribution of NORs makes (AD)_4_ differ largely from the rest of all disomic cottons, which is somewhat in line with the ITS data by Wendel et al. (1995).

### Karyotype of *G. mustelinum*

**Figure 5:**
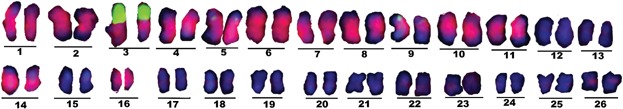
Karyotype analysis of Gossypium mustelinum.

Karyotype analyses of *G. mustelinum* based on dual-FISH were used to identified the location of GISH-NOR (Figure 5), with the super-major GISH-NOR being located at chromosomes 3, while the minor GISH-NORs were located at chromosomes 5 and 9, respectively. Not all A sub-genome chromosomes (red signals) were longer than D sub-genome chromosomes (blue). Chromosomes 12 and 13 of the A sub-genome were actually the same as chromosomes 14 and 16 in length, while chromosomes 1, 2, and 3 of the D sub-genome were longer than chromosome 13 of A sub-genome.

## Discussion

### Size and type of (AD)_4_ GISH-NORs

Previously, we have reported 45S rDNA loci of *G. mustelinum* ((AD)_4_) (Wu et al. 2008). In this experiment, dual-FISH with gDNA and 45S rDNA as probes were at the same location as 45S rDNA and GISH-NOR, suggesting that GISH-NOR were generated by the 45S rDNA in gDNA. The six major 45S rDNA or GISH-NOR signals were distributed on six chromosomes in mitotic metaphase spreads in (AD)_4_, which were same as the other four allopolyploid cotton species (Liu et al. 2005; Song et al. in 2013, Xiao et al. in 2013). Though NOR and GISH-NOR numbers were the same in allopolyploid cotton species, the sizes and sub-genome sites were largely different between (AD)_4_ and other AD species. Firstly, one of the GISH-NORs in (AD)_4_ was huge in size, possibly over 100 times larger than the other two, while the GISH-NORs were relatively similar in the other four AD species. In that case, we could define the large GISH-NOR as a super-major NOR or super GISH-NOR. Secondly, all NORs or GISH-NORs were detected in A sub-genome chromosomes in (AD)_4_, while one of them were detected in the A sub-genome chromosome and two of them in the D sub-genome chromosomes in the other four allopolyploid cotton species. The huge size and abnormal sub-genome NORs were only found in (AD)_4_, and not in any other *Gossypium* species or other plants, including other eukaryotes.

### (AD)_4_ relationships with its diploid progenitors

We noted in this study that the intensities and densities of the hybridization signals to (AD)_4_ with gDNAs of A genome species (A_1_, A_2_ and their wild form A_1-a_) as probes were much stronger and higher than those of the D genome species (D_5_, D_1_, and D_3-d_), which was illustrated by GISH images of both mitotic and meiosis chromosomes (Figures 2–4). Many authors focused on A genome as the female parent(s) of AD cottons (Wendel 1989), and recent phylogenetic analyses (Small et al. 1998; Jiang et al. 1998, 2000; Wright et al. 1998; Liu et al. 2001) suggest a higher evolutionary rate in the D sub-genome than the A sub-genome, which hint that the GISH signals of allopolyploid cotton with D genome species as probe would be weaker than A genome species as probe. These demonstrate that the relationships of A genome are relatively much closer to (AD)_4_ than D genome, which is consistent with early studies (Wendel 1989; Liu et al. 2005).

As the evolutionary rate in the D sub-genome was higher than the A sub-genome in (AD)_4_, the 45S rDNA derived from the diploid D donor evolved faster than that of the diploid A donor, and the 45S rDNA of (AD)_4_ should be similar to the diploid A genome species. Research on sequence data from the ITS and 5.8S rDNA have shown that nearly all rDNA repeats in *G. mustelinum*, roughly 3,800 in total and each being approximately 10 kb in length, have been homogenized to A-like, with the other allopolyploid species having exclusively D genome-like rDNA (Wendel et al., 1995, Wendel 2000). But in this experiment, GISH-NORs were detected only with the gDNA of D genome species as probes, but not A genome species (Figures 2B–D, 4A–D), suggesting that the 45S rDNA of (AD)_4_ was more homologous to D genome species but not A genome species, or that 45S rDNA derived from D genome donors evolved slower than that of A genome.

In most alloployploid plants, the number of 45S rDNA loci equals the sum of that of their progenitors (Wang and Zhang 2000). However, loss of some loci has been observed in several alloployploid species (Vaughan et al. 1993; Leggett and Markand 1995; Snowdon et al. 1997). *G. mustelinum* is an alloployploid species; its related diploid donor species *G. herbaceum* (A_1_) and *G. raimondii* (D_5_) have two or three major 45S rDNA loci (Hanson et al. 1996; Liu et al. 2005), but in *G. mustelinum*, two 45S rDNA loci are minor, while 45S rDNA in the other four allopolyploid *Gossypium* species are major. In the evolution of polyploid species, concerted evolution plays an essential role in the maintenance of sequence homogeneity in multigene families through inter-chromosomal interaction, unequal crossing over (unequal exchange) and gene conversion (Zhang and Sang 1999; Wendel 2000). Therefore, the mechanism of 45S rDNA evolution in *G. mustelinum* ((AD)_4_) may be different than in other allopolyploid *Gossypium* species, and unequal crossing over (unequal exchange) may play a more important role in the concerted evolution of 45S rDNA in *G. mustelinum*. This results in all of the 45S rDNA of the D genome donor being “exchanged” to A sub-genome chromosomes of (AD)_4_, while the 45S rDNA of the A genome donor is transferred to chromosome 3 to form a super-major 45S rDNA loci, and some remaining 45S rDNA becomes the minor 45S rDNA loci in the A sub-genome chromosomes 5 and 9 in (AD)_4_. Traditional cytogenetic research has indicated that DNA content and chromosome length of diploid A genome species are bigger than that of diploid D genome species, so the 13 longer chromosomes of the tetraploid cotton were named the A sub-genome, while the 13 shorter chromosomes were named the D sub-genome. But to our surprise, in this experiment, chromosomes 12 and 13 of the A sub-genome were actually equal to chromosomes 14 and 16 in length, while chromosomes 1, 2, and 3 of the D sub-genome were longer than chromosome 13 of the A sub-genome, which also holds for *G. barbadense* (Wang et al. 2001). Therefore, classifying the longest 13 chromosomes of tetraploid cotton as the A sub-genome, and the shorter 13 chromosomes as the D sub-genome chromosomes as done in traditional cytogenetic and karyotype analysis may not be entirely correct.

Previous experiments have shown that *G. mustelinum* is the sole descendent of one branch of the earliest divergence within the AD lineages (Small et al. 1998). In this experiment, FISH showed that the 45S rDNA size and location of *G. mustelinum* were different than that of other allopolyploid species, while the evolution of DNA and rDNA of *G. mustelinum* conflict with each other. The evolution of *G. mustelinum* may be considerably more complex and dynamic than previously envisioned, and there is much more work to be done on *G. mustelinum*.

## Materials and Methods

### Materials

*Gossypium mustelinum* is a wild disomic polyploid (2*n* = 4*x* = 56) endemic to Brazil. Its accession was provided by Dr. Percival of USDA-ARS, Cotton Germplasm Research Unit in College Station, Texas, and was primarily collected from Brazil in 1988 with its collection number being PS 118. It was used only as the target chromosomes in this study.

The probes used in this study include gDNA extracted from following diploid cottons (2*n* = 2*x* = 26): *G. herbaceum* var. *africanum* (A_1-a_), *G. arboreum* (A_2_), *G. raimondii* (D_5_), *G. davidsonii* (D_3-d_), and *G. thurberi* (D_1_). The A_1-a_ was provided by the Central Cotton Research Institute of Pakistan in 1986, and the three D genome species were also provided by Dr. Percival. Their accession IP numbers are shown in Table1. The accession of A_2_ is a commercial cultivar, namely Shixiya-1 (SXY-1), and was bred in China in the early 1970s. All these plant materials are being maintained in the Wild Cotton Plantation in Hainan Island, China, or in greenhouses at the Cotton Research Institute, Chinese Academy of Agricultural Sciences, Anyang, China. The 45S rDNA probe was a 8.2 kb Xho-fragment of 45S rDNA, which contained almost a full *Arabidopsis* rDNA repeat and was prepared from the plasmid JDH 2-15A, which was kindly provided by Drs. Dai and Wu (Cornell University, Ithaca, NY, USA).

**Table 1:** Probes and blocks used to hybridize to (AD)_4_ somatic cells (SC) and pollen mother cells (PMC)

Probes	(AD)_4_ cells	Blocks	Accessions or material sources
45S	SC	Salmon sperm	Plasmid of JDH 2-15A
A_1-a_	SC	D_3-d_ gDNA	Collected by Pakistan CCRI
A_1-a_	PMC	D_3-d_ gDNA	Collected by Pakistan CCRI
A_2_	SC	D_5_ gDNA; Salmon sperm	A commercial cultivar SXY-1, bred in China
D_5_	SC	A_1-a_ gDNA; Salmon sperm	PI 530898
D_5_	PMC	A_1-a_ gDNA	PI 530898
D_3-d_	SC	Salmon sperm	PI 530809
D_1_	SC	A_2_ gDNA	PI 530765

A_1-a_, *Gossypium herbaceum* var. *africanum*; A_2_, *G. arboreum*; D_5_, *G. raimondii*; D_3-d_, *G. davidsonii*; D_1_, *G. thurberi*.

**Table 2:** Numbers of slides, different images and hybridization signals for each probe to (AD)_4_ FISHs*

Probes	45S	A_1-a_	A_2_	D_5_	D_3-d_	D_1_	Subtotal
Mitotic spreads							
Total slides	32	9	18	77	12	13	161
Interphase cells	100	33	7	12	3	1	156
Premetaphase cells	11	2	7	22	3	0	45
Metaphase cells	32	19	22	102	19	14	208
1 Super-major + 3 minor	1	0	0	0	1	0	2
1 Super-major + 4 minor	3	0	0	3	0	3	9
2 Super-major + 0 minor	1	0	0	1	4	1	7
2 Super-major + 1 minor	0	0	0	19	0	0	19
2 Super-major + 2 minor	1	0	0	11	1	1	14
2 Super-major + 3 minor	4	0	0	24	4	0	32
2 Super-major + 4 minor	22	0	0	44	9	9	84
Meiotic spreads							
Total slides	0	11	0	17	0	0	28
Metaphase I cells	0	23	0	29	0	0	52

* The results of dual-label FISH images were not included.

A_1-a_, Gossypium herbaceum var. africanum; A_2_, G. arboreum; D_5_, G. raimondii; D_3-d_, G. davidsonii; D_1_, G. thurberi.

### Chromosome preparation

The mitotic spread preparation was carried out mainly according to methods described by Wang et al. (1999, 2001a). Seeds were germinated in sands wetted by distilled water at room temperature (20–25 °C). When root tips were 3–5 cm long, they were excised and pre-treated in cycloheximide (25 ppm) for 2 h before fixation in 3:1 (v/v) ethanol:acetic acid fixing solution for 2–24 h. The root tips were incubated in the enzyme mixture for approximately 1.5 h, and were then squashed in 60% acetic acid. Cover slips were removed after freezing in liquid nitrogen, and slides were air-dried. After root tip collection, the seedlings were transplanted to wet sand. Secondary roots were also collected from these seedlings, and were pretreated in the same way described above. Meiotic chromosome spreads were prepared according to the procedures described by Crane et al. (1993) using floral buds.

### DNA extraction and probe labeling

All DNA was isolated from immature leaves of respective accessions using the techniques described by Song et al. (1998). When being used as a probe, gDNA was sonicated to a length of 300–500 bp before labeling. NDA was labeled with DIG-11-dUTP using DIG-High-Prime and Biotion-16-dUTP using the Biotin-Nick-Translation Mix (Hoffmann-la Roche Ltd., Basel, Switzerland), as described by Wang et al. (1999).

### *In situ* hybridization

The procedure for pretreatment, denaturation, hybridization, post hybridization, and detection used in this study was reported with Wang et al. (1999). Slides were pretreated with RNase A (100 g/mL) for 1 h at 37 °C, pepsin solution (100 g/mL) for 30 min at 37 °C, were fixed in 4% freshly depolymerized paraformaldehyde for 10 min, 2× SSC (0.3 M NaCl, 0.03 M citrate at pH 7.0) for 5 min, and were then dehydrated in 70%, 85%, 95%, and 100% ethanol for 2 min each. The probe mix was denatured at 80 °C for 5 min, applied to air-dried slides in a 25 mL volume, and the slides were denatured at 80 °C for 10 min and then incubated overnight at 37 °C. The probe hybridization mixture consisted of 50% formamide, 10% dextran sulfate, 2 × SSC, 5% sodium dodecyl sulfonate (SDS), probe DNA, and unlabelled blocking DNA. Plastic membranes were carefully removed after hybridization. Slides were then washed three times at 37 °C in 2× SSC containing 0.1% SDS for 5 min, and three times at 37 °C in 0.2× SSC containing 0.1% SDS for 5 min. The slides were then blocked for 5 min at 37 °C by 5% bull serum albumin, rhodamine anti-digoxigenin or fuorescence isothiocyanate incubated at 37 °C for 1h. Chromosomes were counter-stained with DAPI or propidium iodide (PI) for 6–10 min. All slides were incorporated into antifading solution Vectashield (Vector Laboratories, Burlingame, CA, USA).

### Fluorescence microscopy and homology designation

The hybridization signals were observed using a fluorescence microscope (Leica MRA2, Wetzlar, Germany or Ziess Axioskop 2 Plus, Jena, Germany). Images were captured by a charge-coupled device (CCD) system and brought together to make the plate using Adobe Photoshop CS2 software. Images were processed using Adobe Photoshop by changing contrast, brightness and color balance in order to ensure that the whole image was processed uniformly.

For chromosomes adorned with FISH signals, homologous relationships within each cell were inferred on the basis of multiple criteria, including the relative positions of FISH signals on chromosomes, the relative chromosome sizes, chromosome morphology, FISH signal sizes and shapes. The NOR or GISH-NOR positions were interpreted according to the view of *G. mustelinum* as a disomic cotton.
